# Proton radiography interpretation with artificial intelligence for treatment deviation detection in proton therapy^☆^

**DOI:** 10.1016/j.phro.2025.100872

**Published:** 2025-11-20

**Authors:** Giuliano Perotti Bernardini, Arthur Galapon, Gabriel Guterres Marmitt, Jeffrey Free, Peter van Ooijen, Johannes Langendijk, Stefan Both

**Affiliations:** Department of Radiation Oncology, University Medical Center Groningen, University of Groningen, the Netherlands

**Keywords:** Proton radiography, Adaptive proton therapy, Deep learning image classification, Proton range uncertainty, Image-based quality assurance, Treatment verification

## Abstract

**Background and purpose:**

Patient setup errors, anatomical changes, and uncertainties in proton range estimation degrade dose conformity in proton therapy. Adaptive proton therapy (APT) mitigates these deviations by monitoring and adjusting treatment plans. Proton radiography (PR) offers direct proton range information, making it a promising method to detect such deviations. In this study, we developed and evaluated an artificial intelligence (AI) PR tool for automated interpretation and classification of treatment deviations.

**Materials and methods:**

Computed Tomography (CT) scans from 32 head-and-neck cancer patients were synthetically modified to simulate setup errors (±2–4 mm), calibration curve errors (±3–5% for fat/soft tissue, ±7–11% for bone), and anatomical changes (±2–12 mm mimicking weight variations). PR simulations were performed using OpenREGGUI to generate integral depth dose (IDD) curves and range shift maps (RSMs) across 260 × 260 mm^2^ PR fields, resulting in 14,503 RSMs. A convolutional neural network (EfficientNet-v2-M) was trained from scratch for multi-label classification. Performance was evaluated on the synthetic dataset and an independent clinical dataset of 22 patients who underwent plan adaptation.

**Results:**

The CNN classified treatment deviations within one second per image. On the synthetic dataset, it achieved 97% precision, 92% recall, 93% F1-score, and 92% F2-score. On the clinical validation set, it maintained high performance metrics: 86% precision, 88% recall, 86% F1-score, and 87% F2-score, demonstrating strong generalization to clinical scenarios.

**Conclusions:**

The AI-enhanced PR tool enables fast, automated detection of treatment deviations in proton therapy, supporting its integration into APT workflows and online plan adaptation for improved quality assurance.

## Highlights

• Deep learning detects treatment deviations from proton radiography in under 1 s.
• Model trained on synthetic data, generalizes robustly to clinical head and neck cases.
• Deviation detection achieved 97% precision for synthetic images and 86% for clinical .

## Graphical abstract

**Figure:**
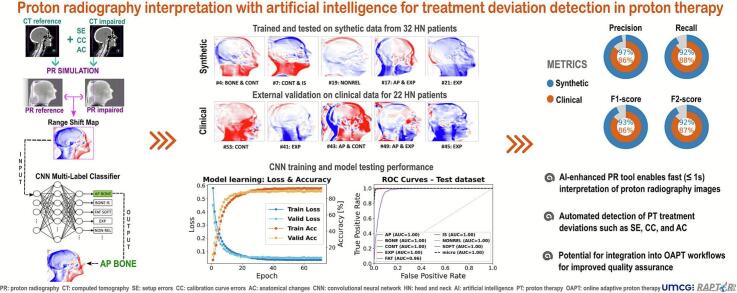

## 1 Introduction

Proton therapy (PT) can deliver more conformal radiation doses compared to conventional photon-based radiotherapy, significantly reducing unwanted exposure to healthy tissues. However, various treatment deviations, including inaccuracies in patient positioning, anatomical changes, organ motion, and proton range uncertainty, may compromise this conformity [1,2]. Thus, having accurate, up-to-date anatomical information and precise proton range estimation immediately before treatment is critical for optimal proton therapy outcomes.

Adaptive proton therapy (APT) addresses these uncertainties by identifying and correcting deviations during treatment. APT uses imaging techniques such as cone-beam computed tomography (CBCT) or in-room CT scans to monitor anatomical variations or setup inconsistencies [[3], [4], [5]]. These detected changes enable clinicians to adapt the original treatment plan or create new plans tailored to daily anatomical conditions, ensuring that clinical goals are consistently met.

Proton radiography (PR) has demonstrated significant potential in reducing range uncertainties by verifying proton range accuracy and patient alignment [[6], [7], [8], [9], [10], [11]]. Recent studies have highlighted its utility as a quality assurance tool for CBCT-based synthetic CTs [[12], [13], [14]], reinforcing its role in online APT workflows. Despite these benefits, integrating PR into routine clinical practice remains challenging due to the complexity of acquisition and interpretation of PR images within clinical workflows.

Convolutional neural networks (CNNs) demonstrated promise in automating complex imaging interpretation and decision-making tasks within proton therapy workflows [15]. In this feasibility study, we evaluated a deep learning classification tool for automatic multi-label identification of treatment deviation sources (including patient positioning errors, anatomical changes, and CT calibration curve errors) to trigger adaptive proton therapy interventions.

## 2 Materials and methods

### 2.1 Patient dataset

We analyzed CT scans from 54 head-and-neck (HN) cancer patients (Databank SFP, ethics approval: 160237) treated with pencil beam scanning proton therapy at the University Medical Center Groningen (UMCG). Due to limited clinical data, CT scans from 32 patients underwent artificial modifications to simulate clinically relevant proton range deviations, including patient setup errors (SE), calibration curve (CC) inaccuracies, and anatomical changes (AC). The remaining 22 clinical patients, who had undergone proton plan adaptations, formed an external validation dataset to evaluate the AI tool’s performance.

### 2.2 Treatment deviations

Treatment deviations were introduced into patient CT scans to mimic realistic uncertainties encountered in clinical proton therapy. Proton radiography was then simulated using these modified and reference CT images, generating impaired and reference PR images, respectively. Proton range shifts resulting from the treatment deviations were quantified by comparing integral depth dose (IDD) curves from impaired and reference PR images. Two-dimensional range shift maps (RSMs) were computed using a least squares approach to determine the optimal offset between IDD curves [7].

Patient setup errors were introduced as shifts in anterior-posterior (AP), and inferior-superior (IS) directions for the PR field accounting for the setup error margin used in our clinic, as well as smaller and larger margins for head-and-neck patients [16]. Clinically relevant setup errors of ± 2 mm to ± 4 mm were generated by sampling random (normal distribution) shifts applied in each direction (AP+, AP−, IS+, IS−), including diagonal shifts.

**Fig. 1:**
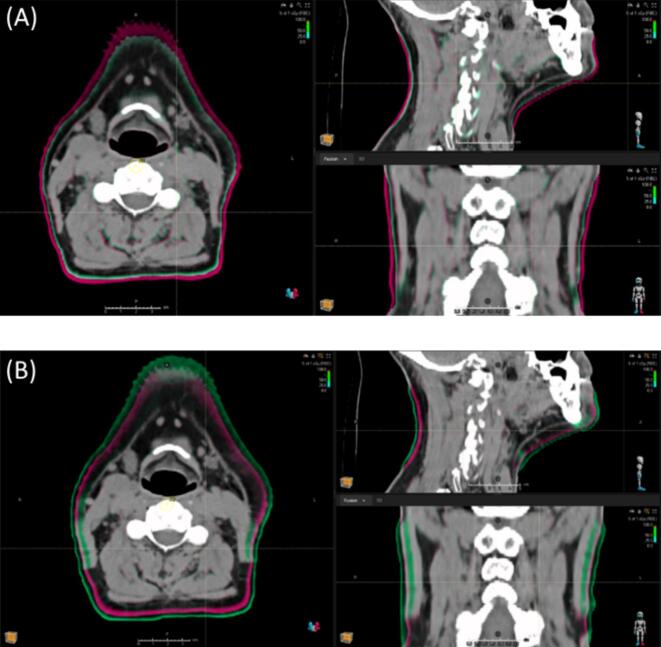
Fusion visualization of original planning CT (magenta) and artificially deformed CT (green). (A) CT deformation representing neck contraction to mimic weight loss. (B) CT deformation representing neck expansion to mimic weight gain.

Anatomical changes were generated with an in-house Anatomical Deformation Algorithm (ADA), using the ANACONDA deformable registration algorithm (RaySearch, Stockholm, Sweden) [17]. ADA simulated realistic anatomical neck variations, reflecting changes typically observed during head-and-neck proton treatments [18]. For each patient, ten artificially deformed CT scans were generated to represent incremental expansions (2 to 12 mm) and contractions (−2 to −12 mm), mimicking patient weight gain or loss. Aside from these localized neck variations, the modified CTs preserved the original patient anatomy, as exemplified in Fig. 1.

Calibration curve errors were produced by randomly perturbing density values for fat, soft tissue, and bone to generate new CT calibration curves. These modified calibration curves were applied to CT images, altering CT numbers to represent realistic inaccuracies [19,20]. Density perturbations (p) were set within clinically relevant ranges: ±3%, ±4%, and ±5% for fat and soft tissue, and ±7%, ±9%, and ±11% for bone. The density modification, based on the original Hounsfield units (HU) value for the corresponding tissue type, is expressed as: d_mod_ = d x (1 + p), with p representing the perturbation percentage. Perturbed curves examples are provided in supplementary Table S1.

### 2.3 Proton radiography simulations

Proton radiography images were simulated using OpenREGGUI, a MATLAB-based platform for adaptive proton therapy [21,22], producing PR images similar to those measured by a Giraffe multi-layer ionization chamber (IBA Dosimetry, Schwarzenbruck, Germany). The algorithm uses direct ray-tracing method, scaling integral depth dose (IDD) curves by water-equivalent thickness derived from CT scans.

Simulations were performed on unperturbed and artificially perturbed CT scans, reflecting realistic treatment deviations. To cover the entire head-and-neck region, thereby addressing various areas where treatment deviations may occur, the dimensions of the proton radiography field were defined as a square field measuring 260 × 260 mm^2^. Proton pencil beams of 210 MeV at a 90° gantry angle and 1 mm spot spacing were used, and a Gaussian kernel (sigma = 3.5 mm) simulated clinically observed range mixing effect [22,23]. These simulated PR images were then used for CNN analysis.

### 2.4 Proton range assessment on range shift maps

To evaluate the proton range accuracy of simulations performed with OpenREGGUI, we retrospectively compared simulated PR images based on unperturbed planning CT scans to in vivo pencil beam PR measurements from seven patients in the CNN testing group. The PR measurements described as range probing quality control (RP-QC) were performed twice during the course of the treatment, and the dose delivered was 1 cGy (RBE)^1^ per RP-QC procedure [9]. These measurements were acquired using a multi-layer ionization chamber (MLIC) detector, delivering 81 proton spots spaced 5 mm apart within a 40 × 40 mm^2^ field at 210 MeV, as previously described by Meijers et al. [9].

OpenREGGUI simulations replicated the measurement setup, matching the geometric conditions and spot positions. Simulated IDD curves were directly compared to measured IDD curves (examples shown in Supplementary Fig. S1). Agreement was assessed by calculating the relative range error (RRE), determined by minimizing the squared differences between the two curves along the depth axis.

**Fig. 2:**
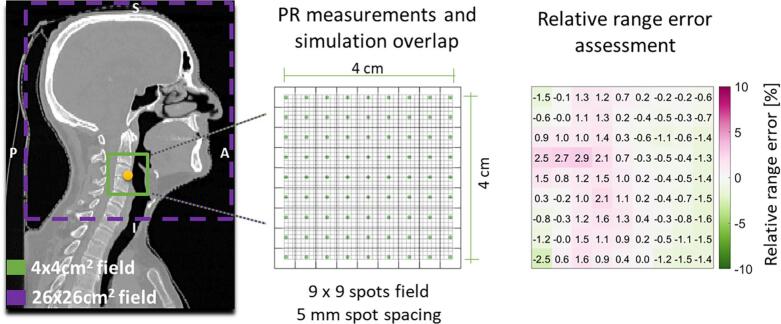
Illustration of proton radiography fields used for validation (example from Patient 3). Simulated PR images (purple area: 260 × 260 mm^2^) were adjusted to match the smaller measured pencil beam PR field (green area: 40 × 40 mm^2^), containing 81 measurement spots around the isocenter (yellow circle). This adjustment allowed a direct comparison between simulated and measured integral depth dose curves, from which a relative range error map was generated.

RRE maps were generated for each patient, highlighting the differences between simulated and measured proton ranges. Fig. 2 illustrates this setup, and results were evaluated against our clinically accepted ±3% proton range uncertainty margin.

### 2.5 Workflow design and implementation

The envisioned clinical workflow for this AI-enhanced PR tool involves comparing a reference image, typically the planning CT, with another image such as a verification CT or CBCT-based synthetic CT, after registration. First, a proton radiography simulation covering the entire head-and-neck region highlights the treatment deviations that may be present during delivery. These deviations can be rapidly interpreted using the CNN presented here. Detecting deviations immediately before treatment can prompt further investigation and suggest plan adaptations in an APT workflow. Additionally, RP-QC measurements assess proton range uncertainty and verify CT number accuracy within the irradiated volume, serving as an independent validation method of the simulated results [13]. These measurements can be performed twice or more frequently, as indicated by PR simulations and triggered by the AI tool findings.

**Fig. 3:**
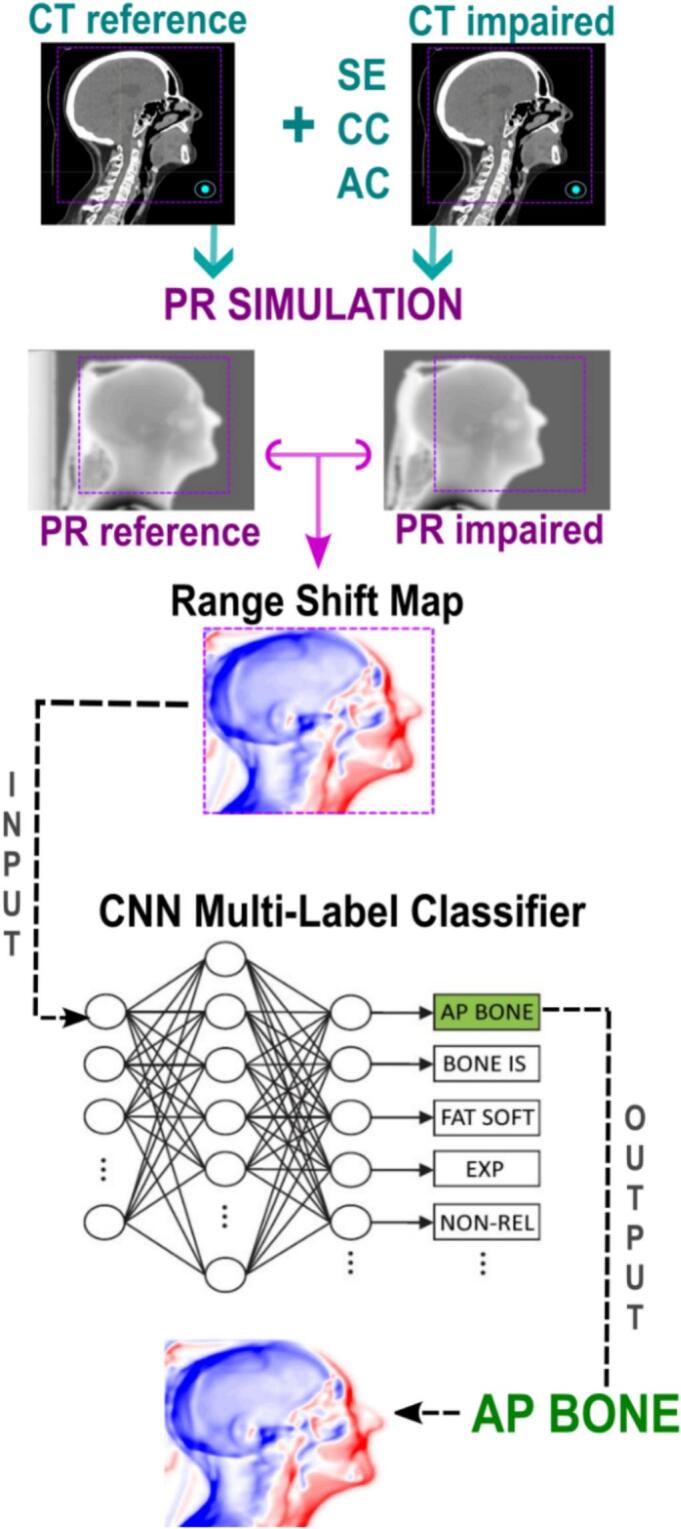
Overview of the workflow design and *in silico* implementation for the AI-enhanced PR tool. Proton radiography simulations generate two-dimensional range-shift maps, which serve as input for a CNN-based 28-class multi-label classifier. The CNN then analyzes each map and assigns labels corresponding to specific treatment deviations identified.

Fig. 3 summarizes the *in silico* workflow used to generate the AI-based PR interpretation tool, starting with proton radiography simulations using original and artificially perturbed CT scans (instead of repeat or synthetic CTs), which are coregistered, followed by the generation of RSM images, and concluding with CNN-based multi-label classification of treatment deviations.

### 2.6 Data interpretation

A convolutional neural network based on the EfficientNet-v2-M architecture [24] was implemented for multi-label classification of two-dimensional RSM images, identifying treatment deviations affecting the proton range (section 2.2). Each RSM image represented single or combined deviations, resulting in 28 distinct classes derived from eight primary categories: anterior-posterior shift (AP), bone errors (BONE), contracted anatomy (CONT), expanded anatomy (EXP), fat tissue errors (FAT), inferior-superior shift (IS), non-relevant deviations (NONREL), and soft tissue errors (SOFT). These 28 classes included both individual categories and their pair-wise combinations (Supplementary Table S2). For instance, “BONE & IS” indicated simultaneous bone CT errors and inferior-superior setup deviations, while “NONREL” captured clinically non-relevant deviations.

EfficientNet-V2-M was selected for its proven efficiency and systematic scalability across diverse image classification tasks, enhancing adaptability to complex medical imaging patterns [[24], [25], [26]]. Its architecture facilitates effective convergence from scratch, useful when pre-training is unavailable or data is limited, without compromising accuracy. Furthermore, EfficientNet models have consistently demonstrated strong performance across medical imaging applications [[25], [26], [27], [28], [29], [30]].

RSM images were channel-normalized with z-score normalization. To mitigate class imbalance, extra samples were created using class-aware augmentation. The EfficientNet-v2-M model was trained from scratch using binary cross-entropy loss, AdamW optimization, and early stopping to prevent overfitting. Model predictions employed sigmoid activation, assigning positive labels when probabilities exceeded 0.5. Multi-label classification performance was assessed using precision, recall, F1-score, and F2-score metrics, and computing receiver operating characteristic (ROC) curves.

The dataset from 54 HN patients was divided into training (22 patients), validation (3 patients), testing (7 patients), and external validation (22 patients) subsets. The CNN-model group (training, validation, and testing) comprised 14,503 RSM images, incorporating predefined treatment deviations (section 2.2) along with additional realistic background variations (±1 mm setup errors, ≤ ±2% calibration curve errors). The external validation dataset included 22 clinically derived RSM images without artificial perturbations (Fig. 6).

### 2.7 External validation for CNN predictions

To evaluate CNN performance on unmodified clinical CT scans, we retrospectively analysed 22 HN patients who underwent plan adaptation. Planning and verification CT scans were co-registered following standard clinical procedures, enabling proton radiography simulations and generation of RSMs to highlight inherent treatment deviations.

These registered CT pairs were reviewed by a medical physicist using the treatment planning system’s fusion visualization mode within a 2000 HU window centred at 0 HU. This blinded assessment identified deviations significant enough to justify plan adaptation, across the entire head-and-neck area covered by proton radiography. Labels from this independent clinical review served as ground truth for evaluating CNN performance.

## 3 Results

### 3.1 Proton range assessment on RSM results

**Fig. 4:**
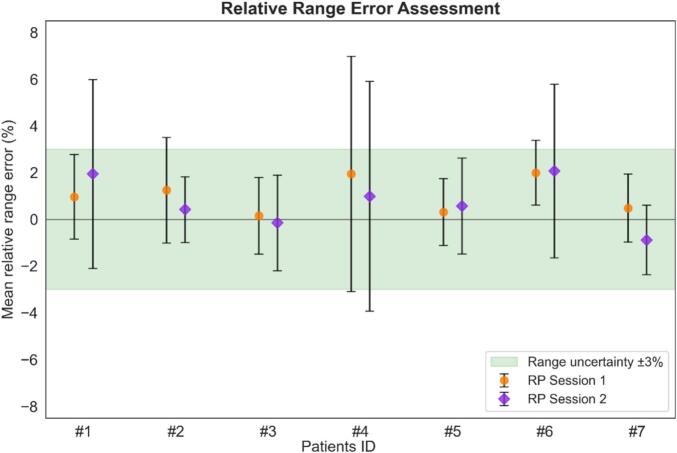
Comparison of mean RRE values and associated variability (1.5 SD, represented by error bars) between measured and simulated IDD curves across RP sessions 1 (orange) and 2 (purple). The ±3% range uncertainty margin is indicated by the green region.

Relative range error maps were generated for all seven patients in the CNN-testing group, with an example provided in Fig. 2. Fig. 4 summarises the mean RRE values and variability (1.5 standard deviations) comparing measured and simulated IDD curves across all patients and measurement sessions. The results consistently showed mean RRE and standard deviations that fell mostly within our clinically accepted ±3% range uncertainty margin.

As shown in Fig. 4, the measured and simulated IDD curves were in close agreement, supporting the range accuracy and reliability of the PR simulations performed with OpenREGGUI within the 40 × 40 mm^2^ region.

### 3.2 Model performance

**Fig. 5:**
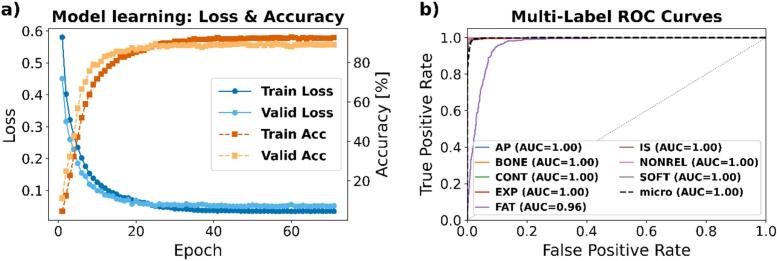
Model performance evaluation. a) Model learning: training and validation loss (blue tones, left axis) and accuracy (orange tones, right axis) versus epoch, overlaid in the same plot, showing stable convergence without evidence of overfitting. b) Multi-label ROC curves: per-class ROC curves (coloured solid lines) and the micro-averaged ROC curve (black dashed) computed on the test set, with AUC reported for each class.

The CNN demonstrated efficient learning from the range shift maps, with training and validation losses decreasing rapidly and accuracies increasing steadily (Fig. 5). No overfitting was observed. The right panel of Fig. 5 shows multi-label ROC curves, where all classes achieved high AUCs, with most near 1.00 and a micro-averaged AUC of 0.998, confirming strong classification ability. Once trained, the model classified each RSM in under one second per image.

**Fig. 6:**
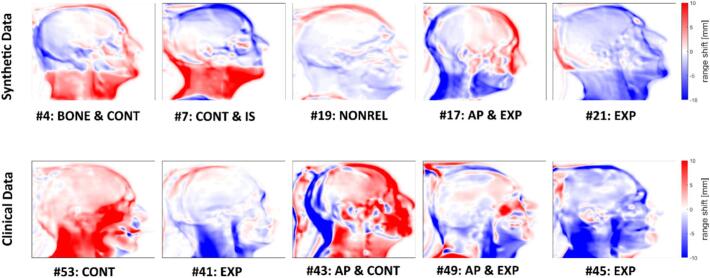
Representative examples of range shift maps from the 28 possible treatment deviation classes. The top row shows RSMs artificially generated by introducing combinations of setup errors, calibration curve deviations, and/or anatomical expansions or contractions around the neck to mimic weight changes. The bottom row shows RSMs derived from PR simulations based on clinically acquired planning and verification CT scans, coregistered without artificial perturbations. Each image displays the patient ID and assigned label. Range shifts are indicated on a scale from –10 mm to 10 mm.

In the CNN-testing dataset, comprising 3,071 RSM images artificially generated from 7 HN patients, the model achieved 97% precision, 92% recall, F1-score of 93%, and F2-score of 92%. When evaluated on an external validation set of 22 HN patients with actual clinical treatment deviations, the CNN maintained high performance, achieving 86% precision, 88% recall, F1-score of 86%, and F2-score of 87%. These results highlighted the model’s ability to generalize from controlled, simulated treatment deviations to complex clinical scenarios.

Representative examples of range shift maps from synthetic and clinical datasets are shown in Fig. 6.

## 4 Discussion

This feasibility study presented an AI-driven proton radiography classification tool designed for rapid and automated identification of proton range uncertainties via range shift maps. The model, developed with synthetic data, demonstrated strong performance when validated on clinical head‑and‑neck cancer cases.

To compensate for limited clinical data, we artificially introduced treatment deviations into planning CT scans, mimicking common clinical uncertainties, including patient misalignment, weight changes, and CT conversion errors. The CNN demonstrated efficient learning dynamics, with training and validation losses decreasing and stabilizing (Fig. 5), and parallel accuracy trends indicating effective model fitting without overfitting. Multi-label ROC curves confirmed strong discriminatory power, with per-class AUC values consistently high (≥0.96) and a micro-averaged AUC of 0.998, reflecting high sensitivity and specificity in detecting treatment deviations. Although trained solely on synthetic RSMs, the model achieved high performance in real clinical cases, demonstrating its ability to generalize and confirming that the synthetic deviations closely replicated clinical scenarios.

For PR simulation validation, simulated IDD curves were retrospectively compared with in vivo MLIC measurements in a 40 × 40 mm^2^ field (Fig. 2). Agreement, assessed via mean relative range error and 1.5 standard deviations, consistently fell within the clinically accepted ±3% range uncertainty margin (Fig. 4), confirming the accuracy of OpenREGGUI simulations in this area. While direct measurement validation at 260 × 260 mm^2^ was not possible because of technical constraints, similar accuracy is expected, and phantom studies by Farace et al. [7,22] support the feasibility of extending this approach.

Acquiring larger-area PR measurements would require sequential couch shifts, impractical due to added time and radiation dose [7]. Therefore, the APT platform OpenREGGUI [22] was used to simulate full-field (260 × 260 mm^2^) PR images representing realistic clinical deviations across the head-and-neck region. While validations have predominantly involved anthropomorphic phantoms [7,21,22], further patient-based evaluations are required.

Although proton radiography is not yet routinely used clinically, this study highlights its potential for detecting treatment deviations [1,10,11,31]. External validation involved PR images simulated from 22 HN patients who underwent plan adaptation, testing the AI tool's ability to identify deviations that would trigger replanning. Registered CT pairs enabled visualization of deviations through PR simulations at plan evaluation. Ground truth labels were established by a medical physicist reviewing planning and verification CTs. Consistent with clinical practice, setup errors and anatomical changes were the most frequently identified deviations (Fig. 6) [3,32]. Calibration curve errors in single-energy CT scans could not be detected by visual inspection alone; thus, no ground truth labels could be assigned for BONE, SOFT, and FAT in the external validation. Consequently, it remains uncertain whether the network generalized well to these specific labels in clinical data.

A key advantage of our AI-enhanced PR tool is rapid inference: each RSM was processed in under one second. Loading the volumetric image and running the PR simulations took ∼44 s, followed by ∼30 s for RSM generation, totalling 75 s per case. Time could be further reduced with hardware or software optimizations. Such speed supports integration into online adaptive proton therapy workflows, ensuring fast detection of treatment deviations is not a bottleneck. For context, current clinical implementations of daily online APT aim to keep the on-couch workflow within ∼30 min [33].

Rapid identification of setup errors could guide immediate patient repositioning using in-room imaging [34]. Detecting inaccuracies in the CT calibration curve could encourage the use of site-specific or patient-specific Hounsfield look-up tables or periodic validation [8]. Furthermore, timely detection of anatomical changes associated with patient weight variations may prompt clinicians to perform dose recalculation and treatment adaptations [3,4,35].

Our study demonstrated the AI tool's sensitivity to subtle anatomical variations. Using an in-house deformation algorithm, localized expansions and contractions simulating weight changes around the neck introduced measurable range shifts. The CNN reliably identified these subtle changes, underscoring its potential for patient monitoring during therapy.

A limitation was that the model did not distinguish between magnitudes of deviations within the same class. Different degrees of anatomical expansion or setup error were labelled with the same label, with no explicit severity indicator despite the RSM intensity range (−10 to 10 mm). Incorporating a secondary regression head or enhancing the architecture for ordinal/continuous prediction could enable magnitude-aware classification [36]. In addition, the model could not identify absolute, patient-specific relative stopping power (RSP) biases that might be common to input images, it primarily detects HU-to-density assignment deviations that produce differences in the simulated PRs.

Scenarios not modelled included rotations and nasal cavity filling. While Scandurra et al. suggested that nasal filling has limited impact with our beam configuration [18], it can be incorporated using methods such as van de Water et al., which assign variable mucosal levels within CTs [37]. This would allow PR simulations across filling states and assessment of their impact, with localized changes likely detectable by the classifier. Extending to such scenarios would enhance the tool’s overall applicability.

This AI-enhanced PR tool is proposed for integration into APT workflows, enabling rapid detection of treatment deviations. As a decision-support tool, it provides valuable information during plan adaptation assessments. Proton radiography simulations are performed across a large PR field using the patient’s updated anatomy before treatment. The AI tool then rapidly identifies and labels treatment deviations, triggering timely clinical interventions, such as acquiring additional in-room imaging, repositioning the patient, or reviewing the CT calibration curve, depending on the detected deviation.

Using these outputs, clinicians can determine the optimal timing and location for in vivo range probing quality control measurements [9]. These targeted measurements, conducted during selected fractions (e.g., during weeks 2 and 4), validate CT number accuracy [13] and confirm the precision of simulated IDDs, supporting PR images reliability.

Finally, a dose recalculation-based plan evaluation, guided by AI-derived insights, determines whether reoptimization or adaptation is warranted. This envisioned workflow ensures that clinical decisions are rooted in patient-specific data, thereby enhancing the precision and adaptability of proton therapy. Deploying the AI tool on a fraction-by-fraction would enable continuous monitoring of inter-fraction deviations, aligning with recent developments in daily APT, where treatment plans are adjusted in real time while the patient remains on the couch [4].

Following a quality assurance assessment of AI-based tools as described by Claessens et al. [38], integrating this AI-enhanced PR tool into automated clinical workflows appears feasible. Such integration would streamline image interpretation, reduce workload, and support online decision-making.

In conclusion, our study demonstrated that training a CNN on synthetic PR simulation data enabled reliable detection of treatment deviations and achieved robust performance in clinical validation. These findings support the potential integration of this approach into online adaptive proton therapy workflows to enhance treatment plan monitoring and decision support.

## CRediT authorship contribution statement

**Giuliano Perotti Bernardini:** Writing – original draft, Investigation, Formal analysis, Conceptualization, Methodology, Visualization, Data curation, Software. **Arthur Galapon:** Data curation, Conceptualization, Writing – review & editing. **Gabriel Guterres Marmitt:** Conceptualization, Writing – review & editing. **Jeffrey Free:** Validation, Writing – review & editing. **Peter van Ooijen:** Writing – review & editing. **Johannes Langendijk:** Resources. **Stefan Both:** Conceptualization, Supervision, Writing – review & editing.

## Declaration of competing interest

The authors declare that they have no known competing financial interests or personal relationships that could have appeared to influence the work reported in this paper.

## Footnotes

1 Here, “cGy (RBE)” denotes the RBE‑weighted absorbed dose (absorbed dose × RBE), following ICRU Report 93 definitions.
