# Supplementary material for: Proton radiography interpretation with artificial intelligence for treatment deviation detection in proton therapy
Source: Phys Imaging Radiat Oncol. 2025 Nov 20;36:100872. doi: 10.1016/j.phro.2025.100872 (PMC12720023; doi:10.1016/j.phro.2025.100872)
Supplement: Supplementary Data 1 [file mmc1.pdf]

Supplementary Materials

[HU]	**-992**	**-976**	**-480**	**-96**	**48**	**128**	**528**	**976**	**1488**	**1824**	**2224**	**2640**	**2832**	**2833**	**3096**
[g/cm^3]	**0.001**	**0.001**	**0.500**	**0.950**	**1.050**	**1.100**	**1.350**	**1.600**	**1.850**	**2.100**	**2.400**	**2.700**	**2.830**	**7.870**	**7.870**
[g/cm^3]	0.001	0.001	0.500	0.903*	1.050	1.100	1.350	1.600	1.850	2.100	2.400	2.700	2.830	7.870	7.870
[g/cm^3]	0.001	0.001	0.500	0.950	1.092†	1.144†	1.350	1.600	1.850	2.100	2.400	2.700	2.830	7.870	7.870
[g/cm^3]	0.001	0.001	0.500	0.950	1.050	1.100	1.472‡	1.744‡	2.017‡	2.289‡	2.616‡	2.700	2.830	7.870	7.870

Supplementary Table S1: Displays a generic reference CT calibration curve (bold), including Hounsfield unit (HU) values and corresponding densities (g/cm^3), alongside examples of perturbed calibration curves: -5% fat (*), +4% soft tissue (†), and +9% bone (‡). Calibration curve errors combining two tissue types were generated by simultaneously perturbing densities of the selected tissues based on the reference calibration curve.

**AP**	**SOFT**	BONE & CONT	FAT & IS
**BONE**	AP & BONE	BONE & EXP	FAT & SOFT
**CONT**	AP & CONT	BONE & FAT	IS & CONT
**EXP**	AP & EXP	BONE & IS	IS & EXP
**FAT**	AP & FAT	BONE & SOFT	IS & SOFT
**IS**	AP & IS	FAT & CONT	SOFT & CONT
**NONREL**	AP & SOFT	FAT & EXP	SOFT & EXP

Supplementary Table S2: Lists the 28 labels derived from the combinations of the eight primary classes ('AP,' 'BONE,' 'CONT,' 'EXP,' 'FAT,' 'IS,' 'NONREL,' 'SOFT'), highlighted in bold. Each RSM was assigned one or two of these classes based on CNN predictions, forming multi-labels such as "SOFT & CONT."

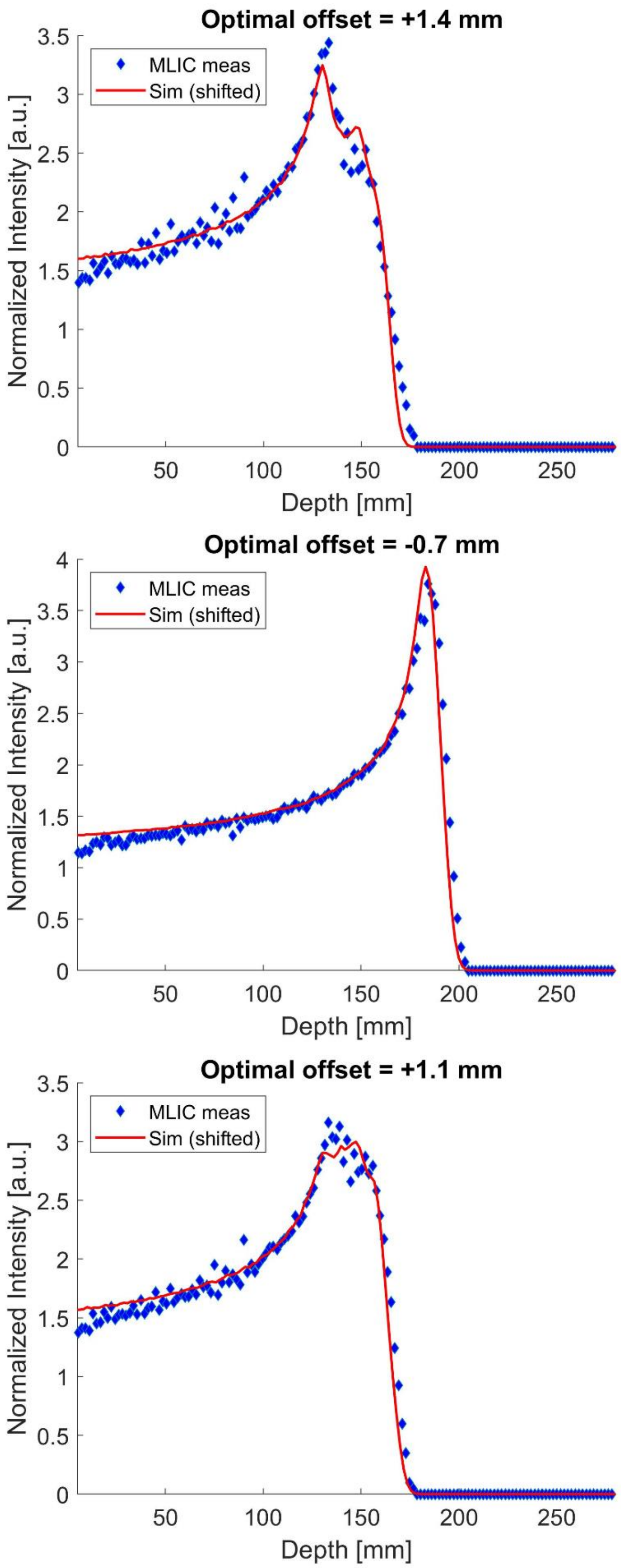

Supplementary Figure S1: Illustrative IDD curves measured with an MLIC and simulated with OpenREGGUI for three pencil beams. Each plot shows both datasets aligned by the optimal offset determined using the least squares method, which corresponds to the spot-specific range error. Negative and positive values indicate that the simulated IDD has a shorter or longer range, respectively, than the measured IDD. Range errors are reported as relative

values (relative range error, RRE) with respect to the water-equivalent path length (WEPL) derived from measurements. WEPL is defined as the difference between the depth of maximum dose in the RP IDD and the maximum depth of a measurement in air for the same proton energy [7].
